# Anti-Correlated Myelin-Sensitive MRI Levels in Humans Consistent with a Subcortical to Sensorimotor Regulatory Process—Multi-Cohort Multi-Modal Evidence

**DOI:** 10.3390/brainsci12121693

**Published:** 2022-12-09

**Authors:** Leighton Barnden, Benjamin Crouch, Richard Kwiatek, Zack Shan, Kiran Thapaliya, Donald Staines, Sandeep Bhuta, Peter Del Fante, Richard Burnet

**Affiliations:** 1 National Centre for Neuroimmunology and Emerging Diseases, Menzies Health Institute Queensland, Griffith University, Southport, QLD 4222, Australia; 2 Nuclear Medicine Department, Royal Adelaide Hospital, Adelaide, SA 5000, Australia; 3 Division of Medical Subspecialities, Lyell McEwin Hospital, Elizabeth, SA 5112, Australia; 4 Thompson Institute, University of the Sunshine Coast, Birtinya, QLD 4575, Australia; 5 Medical Imaging Department, Gold Coast University Hospital, Parklands Drive, QLD 4215, Australia; 6 Endocrinology Department, Royal Adelaide Hospital, Adelaide, SA 5000, Australia

**Keywords:** myelin, regulation, reticular activation system, hypothalamus, amygdala, hippocampus, sensorimotor, subcortical, human, interneurons

## Abstract

Differential axonal myelination synchronises signalling over different axon lengths. The consequences of myelination processes described at the cellular level for the regulation of myelination at the macroscopic level are unknown. We analysed multiple cohorts of myelin-sensitive brain MRI. Our aim was to (i) confirm a previous report of anti-correlation between myelination in subcortical and sensorimotor areas in healthy subjects, (ii) and thereby test our hypothesis for a regulatory interaction between them. We analysed nine image-sets across three different human cohorts using six MRI modalities. Each image-set contained healthy controls (HC) and ME/CFS subjects. Subcortical and Sensorimotor regions of interest (ROI) were optimised for the detection of anti-correlations and the same ROIs were used to test the HC in all image-sets. For each cohort, median MRI values were computed in both regions for each subject and their correlation across the cohort was computed. We confirmed *negative* correlations in healthy controls between subcortical and sensorimotor regions in six image-sets: three T1wSE (*p* = 5 × 10^−8^, 5 × 10^−7^, 0.002), T2wSE (*p* =2 × 10^−6^), MTC (*p* = 0.01), and WM volume (*p* = 0.02). T1/T2 was the exception with a *positive* correlation (*p* = 0.01). This myelin regulation study is novel in several aspects: human subjects, cross-sectional design, ROI optimization, spin-echo MRI and reproducible across multiple independent image-sets. In multiple independent image-sets we confirmed an anti-correlation between subcortical and sensorimotor myelination which supports a previously unreported regulatory interaction. The subcortical region contained the brain’s primary regulatory nuclei. We suggest a mechanism has evolved whereby relatively low subcortical myelination in an individual is compensated by upregulated sensorimotor myelination to maintain adequate sensorimotor performance.

## 1. Introduction

Brain function relies on the integration of information from diverse brain regions. Axons conduct neuronal signals between them and signal conduction speed is regulated by the thickness of the myelin that ensheathes them. The regulation of axonal myelination forms the basis of synchronous intra-brain communication and efficient brain function. This work was motivated by the incidental finding in a clinical T1wSE study of an anti-correlation between sensorimotor and brainstem MRI levels [1], which were a surrogate for myelin.

To confirm this novel relationship, we here refine the methodology of the original study and importantly, apply it to nine independent image-sets from three different cohorts and five MRI modalities. The three cohorts of controls and patients represented 15 years of our MRI research program and offered an ideal extended dataset for the confirmation aspect of the present study. For cohort 1, three MRI sequences were recommended but, with advancing technology and insights, more were added for the second and third cohorts. Strong results were detected in little-used spin-echo MRI which, because it is insensitive to patient-induced magnetic field inhomogeneities, has lower inter-subject variance and, therefore, enhanced statistical sensitivity to human brain tissue variability. The physiological interpretation of variations in the MRI signal is complex, although there is now compelling evidence, outlined below, that myelin contributes strongly to both T1- and T2-weighted MRI.

To our knowledge, only one study quantifies the relative contribution of myelin and iron to the MRI relaxation rates R1 (=1/T1) from spin-lattice interactions and R2* (=1/T2*) from spin–spin interactions, in both grey matter (GM) and white matter (WM) [2]. Elemental concentrations of iron, phosphorus and sulphur in samples of brain tissue were measured with proton-induced X-ray emission and compared with R1 values from Magnetization Prepared RApid Gradient Echo 2 readouts (MP2RAGE), and R2* from multi-echo gradient echo MRI of the samples. Because most brain phosphorus is located in myelin phospholipids, Stuber et al. were able to include iron and myelin concentrations in a linear model to accurately fit R1 and R2* to tissue content. Table 1 lists the contributions of myelin and iron to contrast in R1 (=1/T1) and contrast in R2* (=1/T2*) MRI in grey matter (GM) and white matter (WM) [2].

Only R2* data was available which represent the tissue R2 influenced by local magnetic field inhomogeneities. However, the relative contributions of tissue myelin and iron to contrast should be similar for the actual R2 relaxation times, particularly in WM.

In WM, myelin is a major contributor to MRI contrast (90% for R1, 56% for R2*). Spin-echo (T1wSE and T2wSE) sequences, although seldom used, will respond to myelin levels without the inter-subject variability of patient-induced magnetic field inhomogeneities that affect T2* and bedevil gradient echo images [3]. In cross-sectional clinical studies, T1wSE and T2wSE have been successful in the detection of clinical differences [1,4,5,6].

Our current knowledge of myelination processes derives predominantly from animal studies at the cellular level [7]. The concept of deriving details of macroscopic human myelin regulation from cross-sectional studies is quite new.

We wished to test the hypothesis that sensorimotor myelin levels in humans are regulated by the subcortical nuclei of the reticular activation system (RAS) in the brainstem in concert with the adjacent connected hypothalamus, amygdala and hippocampus. We tested this using T1wSE images from three independent cohorts of healthy controls (HC) and performed regressions between their subcortical and sensorimotor levels. Four other MRI image-sets that respond to myelin levels were also tested: T2wSE, Magnetization Transfer Contrast (MTC) known to respond to macromolecules (mostly myelin in white matter) with a hydration layer which exchanges with free water molecules [8], regional white matter volumes from voxel-based morphometry (see Section 2) that will reflect local myelin levels, and a T1/T2 ratio.

## 2. Materials and Methods

### 2.1. Subject Cohorts and Image Sets

Seven MRI image-sets were analysed from 3 cohorts. All three cohorts included Healthy controls (HC) and myalgic encephalomyelitis/chronic fatigue syndrome (ME/CFS) subjects, although the HC are our primary focus here. Our aim was to test for negative (anti-) correlations between subcortical and sensorimotor white matter regions in the HC of each modality in each cohort.

Ethics approval was obtained for all cohorts—from the Ethics Committee of the Royal Adelaide Hospital (2006 and 2012 cohorts), and the Ethics committees of Griffith University and the Gold Coast University Hospital where scanning was performed (2016 cohort). Informed consent was obtained from all subjects. Because the additional analysis reported here was only a minor extension of the principal SPM processing already performed for all cohorts, and it was performed by the original investigators, no further approval was sought from the relevant Ethics Committees. De-identification of scans preserved subject anonymity.

Ages (mean ± SD years) and genders (f/m) of the three (2006, 2011, 2016) HC cohorts were (33.7 ± 10.3, 34.7 ± 8.4, 43.1 ± 13.7) and (19/6, 10/4, 19/8), respectively.

Five different MRI sequences were analysed here, T1wSE in all 3 cohorts, T2wSE in one cohort, the anatomical O3D FSE and T1wGRE in the 2016 cohort, and MTC in the 2012 cohort. O3D FSE images exhibit the contrast of T2 weighted images and are more commonly known as ‘T2 SPACE’. We call them T2SPACE hereafter. In addition, two image-sets derived from these were analysed: (1) the anatomical T2SPACE images were used to generate relative white matter volume images (see VBM below) and (2), the ratio ‘T1/T2’ which has been reported to respond to myelin levels [9,10,11] was derived using T1wGRE for T1 and T2SPACE for T2. The ‘nAv’ MRI setting in Table 2 was 2 for all T1wSE image-sets and is included because recent (unpublished) work using T1wSE with nAv = 1 did not detect a regression.

### 2.2. Image Processing

SPM12 (www.fil.ion.ucl.ac.uk/spm) was used to perform all voxel-based pre-processing and statistical analysis.

#### 2.2.1. Preprocessing: Spatial Normalization

In order to perform meaningful comparisons of brain structures between individuals and groups, their scans must be ‘spatially normalized’ to the same anatomical ‘space’. Although not perfect, a particular voxel in the MRI image then characterizes the same brain location in all individuals. Anatomical-scan-driven spatial normalization was applied to the T1wSE, T2wSE, MTC, T1GRE and T2SPACE scans. For the 2006 and 2012 cohorts, the anatomical scan was a spoiled gradient-echo (MPRAGE) (TR/TE/flip angle = 5.76 ms/1.9 ms/9°) scan, while for the 2012 cohort it was the T2SPACE ‘optimised 3D fast spin echo’ (Mugler, 2014) scan (Table 2).

The anatomical images were first segmented into GM, WM and CSF using SPM’s unified probabilistic framework [13]. Non-linear spatial normalization of the WM partition was then performed using SPM’s DARTEL toolbox [14] that coregistered the WM partition to a cohort self-template progressively refined over 6 iterations. An additional affine transformation of the final DARTEL WM template to the standard Montreal Neurological Institute (MNI) WM template was computed. The merged deformation for each subject was then applied to the T1wSE, T2wSE, MTC or T1GRE scans after they were coregistered to the reference anatomical scan.

T1/T2 images were generated using a specialised SPM12 toolbox [10] using the 2012 cohort MPRAGE and T2SPACE image-sets.

#### 2.2.2. VBM

The GM and WM partitions were further processed using voxel-based morphometry, VBM [15] to generate MNI-space GM and WM images that encoded regional volume changes associated with the local compression or dilation of the DARTEL non-linear deformation. The VBM white matter images are labelled ‘WM vol’ here.

#### 2.2.3. Preprocessing: Global Values

Global levels for individual T1wSE, T2wSE, MTC, T1GRE and T2SPACE scans were estimated with the validated voxel-based iterative sensitivity (VBIS) method [16] using an inhouse written MATLAB script. For each of these image-sets, a group comparison between HC and ME/CFS was performed using SPM’s proportional scaling. From this result, VBIS extracted the cohort inter-subject variance for each voxel. It then defined a reference region where the voxel variance was below the median voxel variance. The global level for each subject was the mean voxel value in this reference region. An example reference region is shown in [1]. Global levels estimated with the VBIS method and age were then included as nuisance covariates in each SPM statistical model.

In the WM volume analysis, the total WM volume was used as the global.

### 2.3. Regions of Interest (ROIs)

**Figure 1:**
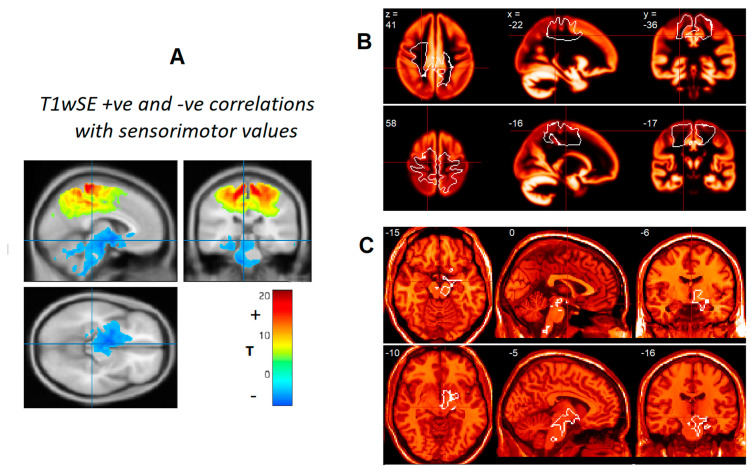
Sensorimotor and Subcortical ROIs. (**A**) Clusters shown as maximum and minimum T value projections from positive (superior) and negative (inferior) voxel-wise T1wSE self-correlations with sensorimotor levels. The threshold for cluster formation was *p* = 0.0001. (**B**) Edges (in white) of the sensorimotor ROI formed by thresholding the positive correlation cluster at T = 7.0 (*p* = 1 × 10^−9^) shown on sections of the grey matter tissue probability map from SPM12. The low probability dark areas within the ROI are white matter (WM), so most of the ROI is either WM or intermediate probability WM. (**C**) Edges of the subcortical ROI formed by thresholding the negative correlation cluster at T = 5.0 (*p* = 2 × 10^−6^) shown on sections of the single subject T1 reference from SPM12. The subcortical ROI includes the neurons and connections of the brainstem cuneiform, dorsal Raphe, pontine and medulla RAS nuclei, the left VTA, posterior hypothalamus, amygdala and hippocampus. Edge voxels are within the ROIs.

Median voxel values sensorimotor WM were derived in a previous T1wSE between-group comparison of HC and ME/CFS subjects in the 2016 cohort [1]. To generate regions optimised for the detection of reciprocal relationships between subcortical and sensorimotor values, we performed a voxel-wise correlation of the 2016 cohort T1wSE images with their sensorimotor medians. Voxel clusters from the negative and positive correlations (Figure 1A) occupied subcortical and sensorimotor areas, respectively. Voxel values in these clusters were highly significant and two ROIs were created from the binarized clusters with a T statistic threshold T = 7.0 (*p* = 1 × 10^−9^) for the sensorimotor ROI (Figure 1B), and T = 5.0 (*p* = 2 × 10^−6^) for the subcortical ROI (Figure 1C). The new sensorimotor WM and subcortical ROIs were used to test the anti-correlation hypothesis in all image-sets.

Figure 1B shows the sensorimotor ROI superimposed on the GM tissue probability map from SPM12. This highlights that although some of the ROI covers GM, it is predominantly in WM areas with low GM probability. Because this ROI will include sulci in individual subjects, we computed the ROI medians to minimise the effect of outlier sulcal voxel values.

### 2.4. Removal of Group, Global and Age Variance before ROI Evaluation

The seven MRI cohorts in Table 2 all contained a group of healthy control (HC) and a group of ME/CFS subjects. For each image-set, a 2-sample SPM statistical comparison of the two groups was performed incorporating global level and age as nuisance covariates. SPM voxel-based statistical analysis models inter-subject variability in each MRI voxel as the sum of the effect of group, the effect of global, the effect of age and a residual free of their influence which we use in our analysis below. It does this by solving the general linear model (GLM) equation for each voxel.

Y_i_ = b_1_X_1_ + b_2_X_2_ + b_3_X_3_ + b_4_X_4_ + e_i_

The inputs to this model are the 3D MRI images Y_i_ and the vectors X_1_, X_2_, X_3_, X_4_ each with 1 value for each subject (i indexes the N subjects, an additional index-labelling voxel location was omitted for Y, b and e). X_1_ and X_2_ are binary vectors defining each group (0 or 1 identifies the group for each subject), X_3_ is the vector of global levels, and X_4_ the vector of ages. Solving the equation yields b_1_, b_2_, b_3_, b_4_ and e_i_ which are 3D matrices with the dimensions of Y. b1 to b4 quantify the components of the cohort variability that depend on the group, global and age. e_i_ describes the residual image content for each subject that is independent of group membership, global values or age and can be used to compute their statistical inference. We applied this GLM approach to all seven image-sets. Note that GLM analysis here of HC and ME/CFS groups was a choice of convenience. The pooled HC and ME/CFS subjects could be divided into any two arbitrary groups for the GLM analysis.

The same approach was taken with the derived WM-volume and T1/T2 image-sets.

### 2.5. Evaluation of Subcortical and Sensorimotor ROI Levels

The SPM GLM method was used here to isolate the variance associated with age, group membership and global signal level and generate images of the residual e_i_ which are free of their influence. We used an in-house MATLAB (The Mathworks Inc., Natick, MA, USA) script to extract the residual image for each subject and compute the median (or mean) within each ROI. Subcortical and sensorimotor values for each subject were analysed for their population correlation using Matlab’s ‘linfit’ which yielded the correlation statistical inference (R^2^ and *p* statistics) and the slope of its linear fit. Before plotting the ROI median MRI signals for each subject, the group mean was added. Here, we limit our attention to the ROI correlations in the HC group.

## 3. Results

Voxel-wise T1wSE regressions of preliminary [1] sensorimotor values yielded large clusters for both positive and negative regressions in the sensorimotor and subcortical areas, respectively (Figure 1A). Corrected voxel and cluster p-values were highly significant. ROIs were generated from the two clusters by binarizing them above a T threshold of 5.0 (*p* < 2 × 10^−6^) for the subcortical ROI and T = 7.0 (*p* < 1 × 10^−9^) for the sensorimotor ROI. The sensorimotor ROI (Figure 1B) extended from the M1 and S1 cortex to deep white matter. The subcortical ROI was contiguous from the amygdala and hippocampus, through the posterior hypothalamus predominantly on the right to the VTA and through RAS nuclei in the midbrain, pons and medulla. The sensorimotor ROI was comparable with the ROI used to generate the preliminary sensorimotor values [1].

The seven image-sets listed in Table 2 with HC and ME/CFS subjects were supplemented with derived WM volume and T1/T2 image-sets. Thus, nine MRI image-sets with HC and ME/CFS subjects were analysed from three cohorts and five different MRI modalities (Table 3). For each image-set, Table 3 lists the characteristics of the *HC* correlations between median values for the subcortical and sensorimotor ROIs. Negative correlations were seen in all spin-echo (SE), the MTC, WM volume and T2SPACE image-sets with statistical inference (*p* < 0.05), and involved all three cohorts. The MTC negative correlation was significant (*p* = 0.01) despite its low (N = 14) sample size. The T1GRE correlation was insignificant. T1/T2 was the exception with a positive correlation (*p* = 0.01).

**Figure 2:**
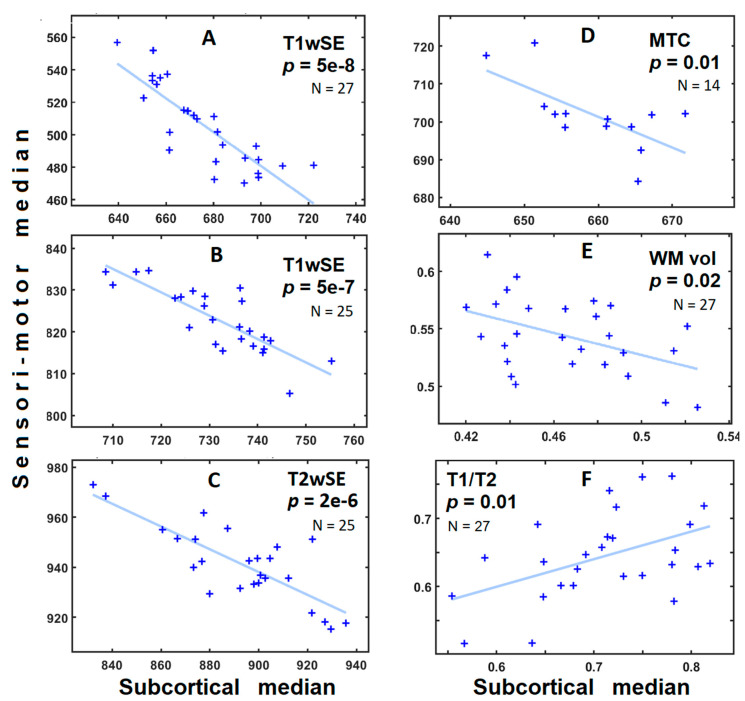
Scatter plots for controls (HC) from 6 image-sets from three cohorts involving five MRI modalities (T1wSE, T2wSE, MTC, WM volume and T1/T2). Each ‘+’ is located by the median MRI value in sensorimotor (y coordinate) and subcortical (x coordinate) regions for an individual subject. Cohort membership is 2016 (**A**,**E**,**F**); 2012 (**D**) and 2006 (**B**,**C**). Blue lines show the linear fit of the HC values. *p* is the probability that the observed HC distribution occurred by chance, and N is the number of HC subjects. HC negative correlations are seen for all image-sets except F. In (**A**,**B**,**C**,**D**) the units derive from the scanner. In (**E**), units are WM volume fraction, in (**F**) units are a ratio. The strongest correlations were from T1wSE (**A**,**B**) with N = 27 and N = 25, respectively, and T2wSE (**C**) with N = 25. The MTC reciprocal correlation was significant despite its low (N = 14) sample size.

Figure 2 shows scatter plots of ROI values for individual subjects for six image-sets with significant negative correlations (*p* < 0.05) and T1/T2. The T1wSE data from the 3 Tesla scanner (2016 cohort) with its advanced 64-channel head-neck receive coil yielded a correlation with (negative) slope near unity. Regressions for other image-sets had lower slopes suggesting that their sensitivity differed for subcortical and sensorimotor myelin. T1/T2 was the exception with a positive slope.

Studies of T1wSE, T2wSE, MTC, T2SPACE and WM volume had a negative correlation with significant statistical inference indicating agreement between these modalities for assessing white matter and myelin. The overall effect size for the correlation between the 2 large ROIs across the image-sets A to E was R = 0.64 (from mean of R^2^ values).

The potential effect of disease was examined by plotting ME/CFS as well as control values (Supplementary Materials, Table S1, Figure S1).

Corresponding regressions for ROI means instead of medians showed similar behaviour. For the 2016 T1wSE image-set the statistical inference after the GLM corrections here (*p* = 5 × 10^−8^) was stronger than with the non-GLM method (*p* = 9 × 10^−4^) [1].

## 4. Discussion

In cross-sectional studies using multimodal human brain MRI images with voxel values sensitive to myelin levels, in 9 independent image-sets, we confirmed the novel anti-correlation between subcortical (brainstem) and sensorimotor myelination reported in 2018 [1]. The inverse relationship was confirmed in two T1wSE, one T2wSE, MTC, T2SPACE and a relative WM volume image-set, all of which respond to myelin levels. For T1/T2, the correlation was positive indicating that this modality is sampling different tissue content, most likely iron (see below).

Lower myelination in the subcortical region was associated with increased myelination in sensorimotor white matter, and vice versa. This study is unique in that it reports unprecedented observations of a myelination relationship across human populations with good reproducibility. The importance in MRI myelination studies of a multimodal approach with MRI measures having consistent, low inter-subject variance was recently emphasized [17].

The subcortical ROI (Figure 1C) used here was created from a voxel-wise negative correlation with sensorimotor levels. It included regulatory nuclei of the brainstem RAS, hypothalamus, amygdala and hippocampus. The hypothalamus, amygdala and hippocampus areas were asymmetric with mostly right-hand presence.

While confirmation of anti-correlated subcortical versus sensorimotor myelination is the primary result of this paper, we propose this is a manifestation of an unreported regulatory process which maintains adequate subcortical–sensorimotor communication in humans. Below we discuss its implications for regulatory mechanisms.

### 4.1. Myelin Regulation

The brainstem RAS influences excitation of the cortex (sensorimotor included) both via projections to the cortex that release excitatory or inhibitory neurotransmitters, and via oscillatory electrical signals that facilitate oscillatory coherence in the cortex [18,19] Both mechanisms may be affected by myelination of the connecting axons. We suggest the following key observations are relevant to the regulation of myelination.

(a) The landmark study of axons from thalamic neurons to somatosensory neurons showed myelination varied to adjust conduction velocity CV so as to yield a constant signal transit time [20,21] and thereby yield isochronicity.
(b) Fisher et al. [22] demonstrated that following the stimulation of different parts of the primary motor cortex (M1) and supplementary motor area (SMA) bilaterally, connections showed a high degree of convergence in reticulospinal neurons of the pontomedullary reticular formation allowing them to integrate information from across the motor areas of the cortex. The same neurons also receive converging sensory inputs from visual, auditory, cutaneous, proprioceptive, and vestibular systems—references in [22]. Thus, their output reflects both central and peripheral activity consistent with the afferent requirements of RAS excitatory neurons.
(c) Connections with the highest myelin content have been reported in regions related to motor and sensory functions [23]. A large proportion of the myelin in the sensorimotor cortex is found along the axons of short-range inhibitory neurons [24].

### 4.2. Regulation of RAS–Sensorimotor Myelination

Bidirectional RAS–sensorimotor communication is critical to appropriate cortical excitation and effective brain function. We assume that there is little lifetime adaptive change in the myelination of the RAS but that the variation observed here (*x* axis in Figure 2 plots) reflects normal human variability. Possible drivers of reciprocal myelination are (i) a requirement to match the excitatory RAS-to-cortex conduction velocities to the positive feedback cortico-reticular conduction velocities, or (ii) the need for coherence in the circuits involving the RAS—intralaminar thalamus—inhibitory interneuron oscillators. To yield isochronicity, a slower conduction velocity associated with lower RAS myelination would require the upregulation of inhibitory interneuron axon myelination and conduction velocity, and vice versa. Given the observed high myelin content of inhibitory interneurons [24,25] and the evidence for a relationship between these oscillators and RAS oscillators [18], option (ii) may predominate, although both could contribute. Inhibitory interneurons which exhibit the highest levels of myelination [24,25] are a likely location for myelin regulation.

Our T1wSE observations show considerable inter-subject variation in sensorimotor myelination (*y* axis in Figure 2 plots). Although a uniform relative difference in sensorimotor myelination in individuals may affect the average conduction velocity, it need not affect the isochronicity of their circuits, the impairment of which could remain hidden from most observers. Indeed, the fact that increased sensorimotor myelination in ME/CFS [1] does not appear to improve sensory or cognitive performance suggests isochronicity is uncoupled from conduction velocity, and isochronicity is reduced in that condition.

### 4.3. Generation of the Two ROIs

Sensorimotor WM levels from an earlier study [1] were used here as a reference to generate two new ROIs optimised for the detection of reciprocal subcortical versus sensorimotor relationships. Two clusters of voxels, one with negative and one with positive correlations, yielded the two ROIs that were used here in the analysis of all image-sets. Positive correlations were only seen in the sensorimotor region (Figure 1A).

Further insights may result by repeating the analysis with alternative subcortical and sensorimotor ROIs, such as the ‘brainstem GM’ and ‘reticulated GM’ isolated by [26], or published atlases.

Our use of self-correlation to generate ROIs optimised for the detection of reciprocal relationships is novel, and perhaps indicative of the paucity of research with low-noise clinical MRI datasets and reciprocal correlations between different regions of the same image-set. A similar approach using self-localization has been reported in fMRI [27].

### 4.4. Advantage of GLM Methodology

Here, we used the SPM general linear model residual from a group comparison (e_i_ in Section 2.4) to exclude variance associated with group membership, age and global signal level and thereby isolate intrinsic brain variability. Only global level variance was excluded in the original method [1]. Variations in average or ‘global’ MRI levels which derive from magnetic field differences associated with individual head anatomy, and position in the scanner, can obscure more subtle variations of interest. The new T1wSE inverse relationship was statistically stronger and, when we applied the same method to 6 additional MRI image-sets sensitive to myelin levels, all exhibited a reciprocal brainstem versus sensorimotor relationship which was significant in all but one case. Although the SPM group comparisons necessary for the evaluation of residual variance were here performed between ME/CFS and HC groups, any arbitrary division of the pooled subjects should yield similar residuals and brainstem versus sensorimotor correlations.

### 4.5. T1/T2 Positive Correlation

T1/T2 images were derived from the ratio of T1GRE (MPRAGE) and T2SPACE images. T2SPACE values decrease with increasing myelination and increasing iron levels. In GM 81% of T2 contrast derives from iron, and in WM it is 44% with myelin contributing to the rest [2]. Thus iron-rich basal ganglia exhibited the highest levels of T1/T2 [11]. Because 1/T2 is non-linearly dependent on T2 and therefore iron levels, it is likely that T1/T2 is responding more strongly to iron than myelin and yields the observed positive correlation between subcortical and sensorimotor levels (Figure 2F).

### 4.6. Spin-Echo MRI Imaging

#### 4.6.1. Myelin or Iron?

Post-mortem studies have established that in WM 90% of R1 (=1/T1) contrast derives specifically from myelin, the remainder from iron [2]. In GM, R1 contrast derived from 64/36% myelin/iron. However, these measures are derived from samples of the sensorimotor and visual cortex and may be different in the brainstem. In our subcortical ROI, the RAS nuclei and the VTA posterior hypothalamus, amygdala and hippocampus can be regarded as GM. It is possible that iron contributes to the T1wSE variance seen in the subcortical ROI here, although brain iron levels were found to be lowest in the brainstem (Ramos et al., 2014). For R2 (=1/T2), iron generates 81% of image contrast in GM and 44% in WM, although the RAS was not evaluated [2].

#### 4.6.2. Spin-Echo Image Properties

T1wSE and T2wSE have the little-appreciated advantage, after correction for individual global differences [16], of small inter-subject variance [28] which is critical in cross-sectional studies [17]. The absence of T1wSE and T2wSE acquisitions in clinical cross-sectional studies to date appears to be a serious omission, particularly in conditions involving white matter.

The detection of clinical T1wSE correlations at 1.5T [4,5,6] motivated us to acquire optimum T1wSE (no turbo) in our 3T study despite an acquisition time of nearly 9 min. This produced group differences with very strong statistical inference [1] and reciprocal correlations that were enhanced here. The added advantage of not acquiring with a turbo sequence could not be resolved from parallel advantages from the increased MRI field strength (3T) and the 64-channel head-neck receive coil [29].

#### 4.6.3. T2SPACE

The ‘Optimised 3D Fast Spin-Echo’ MRI sequence, called ‘T2SPACE’ by Siemens, yields the contrast of T2 images (grey matter signal > white matter > CSF) and has spin-echo advantages. However, the variable flip angle involved gives the images some T1 relaxation time dependence (Mugler, 2014) and their response to myelin levels is not clear. Their negative subcortical vs. sensorimotor correlation (Table 3) had a weaker statistical inference than the standard T2wSE images.

#### 4.6.4. Improved Anatomical Support

Although our use of T2-SPACE as anatomical images was unorthodox, their spin-echo characteristics [12] minimize any distortion, particularly in the brainstem, that may occur with conventional MPRAGE anatomical images due to magnetic field inhomogeneities. Unorthodox but critical to the fidelity of brainstem spatial normalization, was our use of the WM instead of GM probability maps in the DARTEL non-linear spatial normalization process [14].

#### 4.6.5. Effect of Disease

Each healthy control cohort analysed here was accompanied by a cohort of myalgic encephalomyelitis/chronic fatigue syndrome (ME/CFS) subjects. In Supplementary Materials, the same analysis was applied to the ME/CFS cohorts and results are shown in Table S1 and Figure S1. Data overlapped for each modality and similar regressions were detected indicating that the altered myelination in ME/CFS [1] did not affect the sensorimotor—subcortical anti-correlation mechanism.

### 4.7. T1wGRE (MPRAGE)

T1wGRE image levels, although primarily responsive to T1 failed to show any correlation. This suggests their high levels of inter-subject noise related to local, subject-specific magnetic field inhomogeneities and their shorter acquisition times renders them unable to detect a variation in inter-subject myelination.

### 4.8. Artifacts from Scanner Differences

A clear instrumentation difference between the 1.5T scanner (2006, 2011 cohorts) and the 3T scanner (2016 cohort) was the receiver coil: a basic birdcage coil for 1.5T versus a 64-channel head-neck coil for 3T. We asked whether the 3T coil with its depth-dependent sensitivity [29] might introduce an inverse sensorimotor vs. subcortical correlation. We dismissed this concern because a T1wSE image-set was available at both 1.5T and 3T and the correlation was inverse and statistically strong for both (Figure 2A,B).

### 4.9. Limitations

Spatial resolution and coregistration limitations mean the brainstem ROI may not exclude all non-RAS descending corticospinal or ascending medial leminiscus and associated tracts. This makes it difficult to be sure that RAS axonal myelination dominates population variance in the brainstem ROI. Similarly, iron may contribute to the observed RAS variability, although brainstem iron levels are lower than elsewhere [30]. Resolution of this uncertainty may require high resolution cross-sectional studies with MRI sequences that specifically measure myelin [31] and/or susceptibility weighted imaging with good iron contrast [32]. However, such imaging often employs sophisticated multi-step modelling, and associated increases in intra- and inter-subject variance may obscure the relationships reported here.

## 5. Conclusions

Because human myelination and the human brainstem RAS are so difficult to study, observation in a human population of factors that characterise RAS myelination and its correlation with cortical myelination is of novel importance. The novel anti-correlated brainstem-sensorimotor myelination reported here across three cohorts and six MRI modalities confirm a strong association between the two regions and suggest an important, as yet unreported, regulatory mechanism. We propose that the requirement for coherence in the RAS—intralaminar thalamus—inhibitory interneuron oscillator circuit may stimulate inhibitory interneurons to regulate sensorimotor myelination to yield circuit oscillatory coherence in the human population, and thereby produce the observed inverse myelination relationship. As more is learned about regional oligodendrocyte and myelination variability, consideration of this inverse brainstem–cortical relationship may provide important input to the creation of an integrated global myelination narrative.

## Figures and Tables

**Table 1:** Myelin and Iron contributions to R1 and R2* MRI contrast in GM and WM [2].

	R1 (=1/T1)		R2* (=1/T2*)	
	Myelin	Iron	Myelin	Iron
GM	64%	36%	19%	81%
WM	90%	10%	56%	44%

**Table 2:** MRI settings for seven healthy control image-sets across 3 cohorts and 5 MRI modalities. Each image-set is labelled by its year of acquisition. MRI image ‘O3D FSE’ refers to ‘Optimised 3D Fast Spin-Echo’ [12] called ‘T2 SPACE’ by Siemens. Gradient echo T1wGRE is also known as MPRAGE. N is number of HC subjects. The 2006 and 2012 cohorts were acquired on the same scanner.

MRI	Cohort	N	Tesla	TR, TE, Flip Angle	nAv	Voxel Size	Scan Time
Image-Set				(ms/ms/degrees)		X Y Z mm	min:s
T1wSE	2016	27	3.0	600/6.4/90	2	0.86 0.86 3.0	8:52
O3D FSE	2016	27	3.0	3200/563/variable	1	0.88 0.88 0.90	5:44
T1wGRE	2016	27	3.0	2400/1.81/8	1	1.0 1.0 1.0	3:13
T1wSE	2012	13	1.5	600/15/90	2	0.82 0.82 3.0	9:10
MTC *	2012	14	1.5	600/15/90 *	2	0.82 0.82 3.0	6:08
T1wSE	2006	25	1.5	600/15/90	2	0.82 0.82 3.0	9:10
T2wSE	2006	25	1.5	4000/80/90	1	0.86 0.86 3.0	4:24

* T1wSE with off-resonance excitation.

**Table 3:** For nine *Healthy Control* MRI image-sets from 3 cohorts, correlations between subcortical and sensorimotor ROI medians. Correlation statistical inference *p*, R^2^ and linear fit *slope =* Dsensorimotor/Dsubcortical are listed.

*MRI Modality*	*Cohort*	*N*	*p*	R^2^	*Slope*
T1wSE	2016	27	5 × 10^−8^	0.69	−1.0
T1wSE	2012	13	0.002	0.38	−0.60
T1wSE	2006	25	5 × 10^−7^	0.66	−0.56
T2wSE	2006	25	2 × 10^−6^	0.62	−0.46
MTC	2012	14	0.01	0.39	−0.81
T1GRE	2016	27	0.99	0.04	+0.001
T2SPACE	2016	27	0.04	0.12	−0.18
WM volume	2016	27	0.02	0.18	−0.48
T1/T2	2016	27	0.01	0.20	+0.41

## Data Availability

Data unavailable.

## Supplementary Materials

The following supporting information can be downloaded at: https://www.mdpi.com/article/10.3390/brainsci12121693/s1, Figure S1: Scatter plots for both HC (blue) and ME/CFS (red) from 6 image-sets from three cohorts involving five MRI modalities (T1wSE, T2wSE, MTC, WM volume and T1/T2). Each ‘+’ is located by the median MRI value in sensorimotor (y coordinate) and subcortical (x coordinate) regions for an individual subject. Cohort membership is 2016 (A,E,F); 2012 (D) and 2006 (B,C). The linear fit of the HC values (blue) and ME/CFS (red) is shown. p is the probability that the observed distribution occurred by chance and N is the number of subjects for (HC, ME/CFS). HC negative correlations are seen for all image-sets except F. In A, B, C and D the units derive from the scanner. In E, units are WM volume fraction, in F units are a ratio; Table S1: For nine MRI image-sets from 3 cohorts, correlations between subcortical and sensorimotor ROI medians for Healthy Control (HC) and ME/CFS (ME) cohorts. N is the cohort size. Correlation statistical inference p, R2 and linear fit slope = ∆sensorimotor/∆subcortical (not shown if *p* > 0.05) are listed.
