# Supplementary material for: Anti-Correlated Myelin-Sensitive MRI Levels in Humans Consistent with a Subcortical to Sensorimotor Regulatory Process—Multi-Cohort Multi-Modal Evidence
Source: Brain Sci. 2022 Dec 9;12(12):1693. doi: 10.3390/brainsci12121693 (PMC9776387; doi:10.3390/brainsci12121693)
Supplement: Supplementary file 1 [file brainsci-12-01693-s001.zip › brainsci-2088431-Supplementary Material.pdf]

Anti-correlated myelin-sensitive MRI levels in Humans
consistent with a Subcortical to Sensorimotor Regulatory Process.
Multi-cohort multi-modal Evidence

Supplementary Material

---

Each healthy control cohort reported in the main paper was a control cohort in a study of myalgic encephalomyelitis / chronic fatigue syndrome (ME/CFS). The sensorimotor versus subcortical correlation was also performed in the ME/CFS and is reported in Table S1 and plotted in Fig S1. For T1wSE, statistical inference for ME/CFS is comparable with HC in the 2016 cohort, stronger in the 2012 cohort (N = 40 vs 13), and weaker in the 2006 cohort. MTC and WM volume benefited from larger N to yield stronger statistical inference, but comparable for T2wSE and T1/T2. T1GRE remained insignificant.
The regulatory mechanism proposed in the main paper appears unaffected in ME/CFS.

Table S1
For nine MRI image-sets from 3 cohorts, correlations between subcortical and sensorimotor ROI medians for Healthy Control (HC) and ME/CFS (ME) cohorts. N is the cohort size. Correlation statistical inference $p$, $R^2$ and linear fit *slope* = Δsensorimotor/Δsubcortical (not shown if $p > 0.05$) are listed.

| | | *N* | | *p* | | $R^2$ | | *slope* | |
|---|---|---|---|---|---|---|---|---|---|
| *MRI modality* | *cohort* | *HC* | *ME* | *HC* | *ME* | *HC* | *ME* | *HC* | *ME* |
| T1wSE | 2016 | 27 | 43 | 5e-8 | 3e-7 | 0.69 | 0.46 | -1.0 | -0.75 |
| T1wSE | 2012 | 13 | 40 | 0.002 | 2e-5 | 0.57 | 0.38 | -0.60 | -0.62 |
| T1wSE | 2006 | 25 | 25 | 5e-7 | 0.2 | 0.66 | 0.04 | -0.56 | -0.21 |
| T2wSE | 2006 | 25 | 25 | 2e-6 | 0.001 | 0.62 | 0.35 | -0.46 | -0.31 |
| MTC | 2012 | 14 | 40 | 0.01 | 0.002 | 0.39 | 0.22 | -0.81 | -0.40 |
| T1GRE | 2016 | 27 | 43 | 0.99 | 0.15 | 0.04 | 0.028 | +0.001 | +0.08 |
| T2SPACE | 2016 | 27 | 43 | 0.04 | 0.9 | 0.12 | 0.0 | -0.18 | +0.006 |
| WM volume | 2016 | 27 | 43 | 0.02 | 0.003 | 0.18 | 0.17 | -0.48 | -0.38 |
| T1/T2 | 2016 | 27 | 43 | 0.01 | 0.01 | 0.20 | 0.12 | +0.41 | +0.22 |

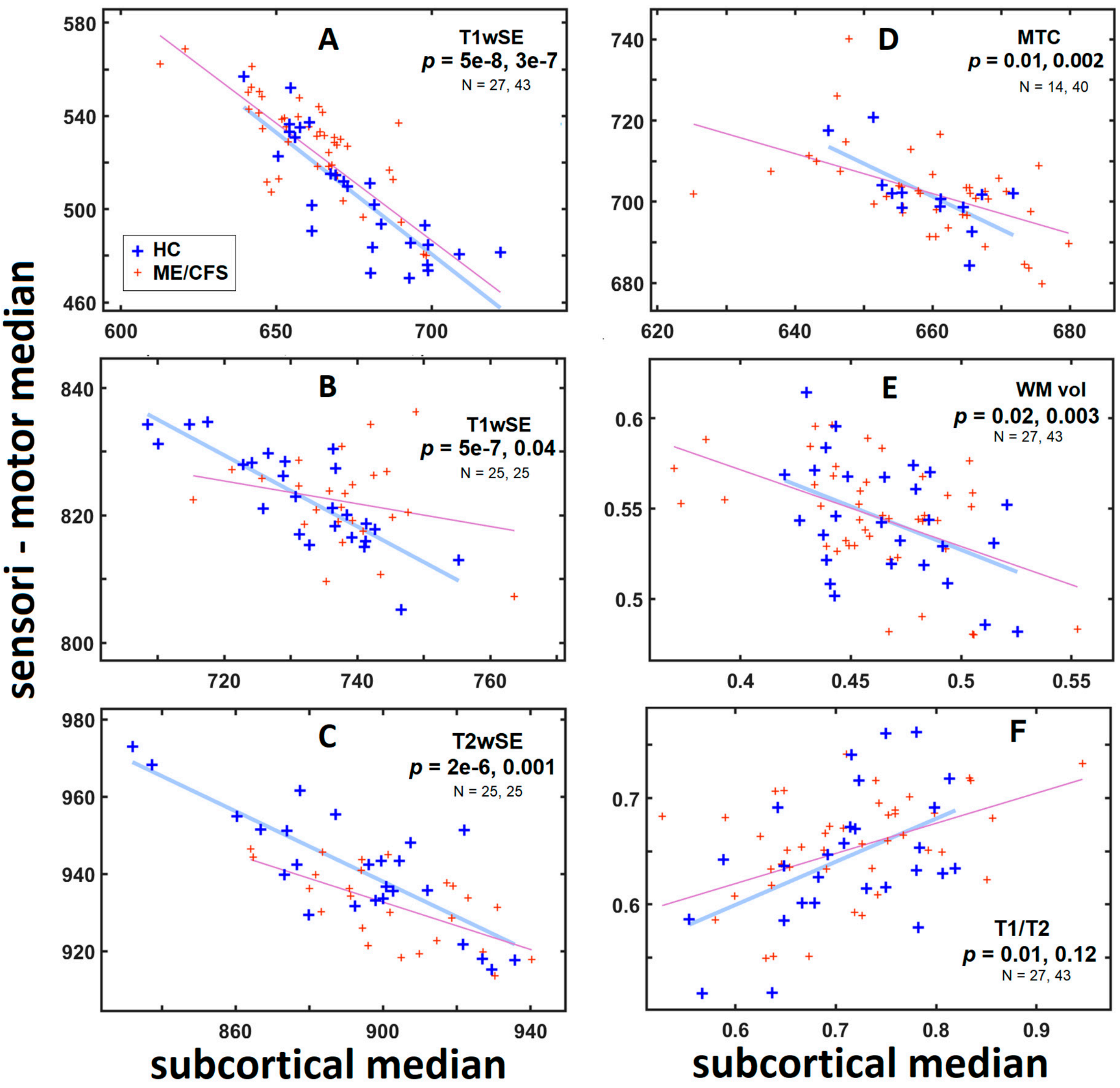


Figure S1

Scatter plots for both HC (blue) and ME/CFS (red) from 6 image-sets from three cohorts involving five MRI modalities (T1wSE, T2wSE, MTC, WM volume and T1/T2). Each '+' is located by the median MRI value in sensorimotor (y coordinate) and subcortical (x coordinate) regions for an individual subject. Cohort membership is 2016 (A,E,F); 2012 (D) and 2006 (B,C). The linear fit of the HC values (blue) and ME/CFS (red) is shown. *p* is the probability that the observed distribution occurred by chance and N is the number of subjects for (HC, ME/CFS). HC negative correlations are seen for all image-sets except F. In A, B, C and D the units derive from the scanner. In E, units are WM volume fraction, in F units are a ratio.
